# Knockout Mouse Models for Peroxiredoxins

**DOI:** 10.3390/antiox9020182

**Published:** 2020-02-22

**Authors:** Young Jae Lee

**Affiliations:** Department of Biochemistry, College of Medicine, Gachon University, Incheon 21999, Korea; leeyj@gachon.ac.kr

**Keywords:** peroxiredoxin, peroxides, reactive oxygen species, knockout mouse, animal model

## Abstract

Peroxiredoxins (PRDXs) are members of a highly conserved peroxidase family and maintain intracellular reactive oxygen species (ROS) homeostasis. The family members are expressed in most organisms and involved in various biological processes, such as cellular protection against ROS, inflammation, carcinogenesis, atherosclerosis, heart diseases, and metabolism. In mammals, six PRDX members have been identified and are subdivided into three subfamilies: typical 2-Cys (PRDX1, PRDX2, PRDX3, and PRDX4), atypical 2-Cys (PRDX5), and 1-Cys (PRDX6) subfamilies. Knockout mouse models of PRDXs have been developed to investigate their in vivo roles. This review presents an overview of the knockout mouse models of PRDXs with emphases on the biological and physiological changes of these model mice.

## 1. Introduction

**Figure 1:**
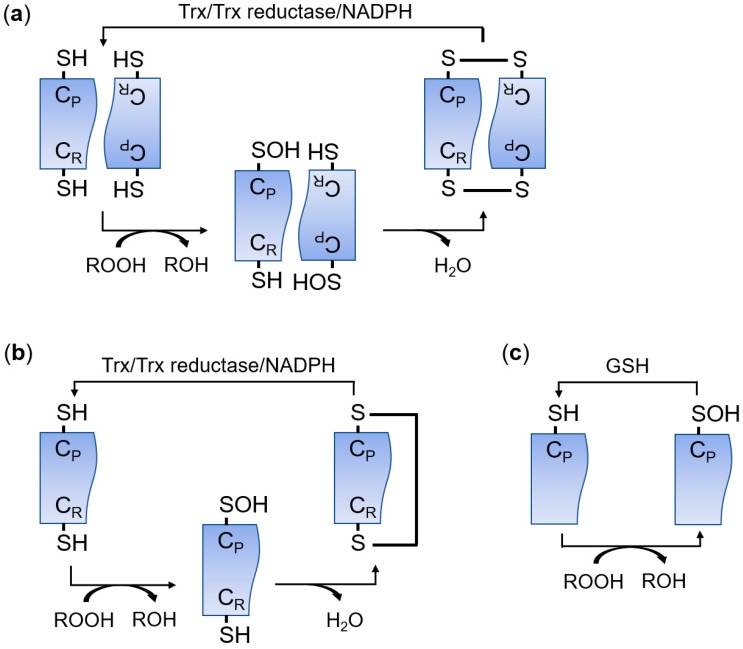
Catalytic cycle of typical 2-Cys (**a**), atypical 2-Cys (**b**), and 1-Cys (**c**) peroxiredoxins (PRDXs). C_P_, peroxidatic cysteine; C_R_, resolving cysteine; GSH, glutathione; ROOH, peroxide; Trx, thioredoxin.

The peroxiredoxin (PRDX) family has peroxidase activity to remove peroxides, including hydrogen peroxide (H_2_O_2_), organic hydroperoxides, and peroxynitrite [1,2]. PRDXs are widely distributed in almost all organisms and there are more than 3500 members in this protein family [3]. PRDXs are classified into three subfamilies (typical 2-Cys, atypical 2-Cys, and 1-Cys) based on the number and location of the active cysteine residues and the type of disulfide bonds produced during the catalytic reaction (Figure 1) [4,5,6,7,8]. Typical 2-Cys PRDXs function as a homodimer [7]. Peroxides oxidize the conserved peroxidatic cysteine (C_P_) in typical 2-Cys PRDXs, and then the oxidized cysteine sulfenic acid residue in a subunit forms an intermolecular disulfide bond with the resolving cysteine (C_R_) in the other subunit [7]. Finally, the intermolecular disulfide bond is reduced by the thioredoxin (Trx)/Trx reductase/NADPH system [7]. Atypical 2-Cys PRDXs remove peroxides using the same mechanism as 2-Cys PRDXs except that atypical 2-Cys PRDXs form an intramolecular disulfide bond between C_P_ and C_R_ within a PRDX molecule [9]. 1-Cys PRDXs have only a C_P_ residue and the oxidized C_P_ is reduced by glutathione (GSH) instead of Trx [10]. According to the PeroxiRedoxin classification indEX (PREX) database that classifies PRDXs based on structural information around active sites, PRDXs are also divided into six subfamilies: AhpC-Prx1, BCP-PrxQ, Tpx, Prx5, Prx6, and AhpE [11,12]. In mammals, there are six PRDX members (PRDX1–PRDX6) [5]. PRDX1–PRDX4 are members of the typical 2-Cys or AhpC-Prx1 subfamily [5,12]. PRDX5 is a member of the atypical 2-Cys or Prx5 subfamily, and PRDX6 is classified into the 1-Cys or Prx6 subfamily [5,12]. Mammalian PRDXs are widely distributed in cells and perform various biological functions. PRDX1 is present in the nucleus and cytosol; PRDX2 and PRDX 6 are in the cytosol; PRDX3 is in the mitochondria; PRDX4 is in the endoplasmic reticulum (ER) and the cytosol; and PRDX5 is in the cytosol, peroxisomes, and mitochondria [8,13].

As genetically modified mouse (GEM) models, knockout mice are useful to investigate the roles of a gene. In the case of mouse *Prdx* genes, several knockout mouse strains targeting each *Prdx* gene have been generated by independent research groups [14]. These knockout mice provide useful information that is hard to obtain from other experiments. The present review summarizes the properties of *Prdx*-knockout mouse models and focuses on the biological and physiological changes of these model mouse strains.

## 2. PRDX1

### 2.1. Genetics and Knockout Mouse Strains

PRDX1 is a ubiquitously expressed nuclear and cytosolic peroxidase protein and is a member of the typical 2-Cys PRDX subfamily [7,13]. PRDX1 is involved in tumor suppression [15,16,17,18], inflammation [13,19,20,21,22,23,24,25,26], apoptosis [19,27,28], atherosclerosis [13,21], and molecular chaperoning [29,30]. According to the mouse Ensembl database, the *Prdx1* gene is located on mouse chromosome 4 and there are five alternative forms of *Prdx1* protein-coding transcripts [31]. *Prdx1*-knockout mouse strains have been generated by the homologous recombination [15,32] or gene trap [17,33] approaches. No *Prdx1*-knockout mouse strain with conditional potential has been reported, although the International Mouse Phenotyping Consortium (IMPC) has embryonic stem (ES) cell clones that possess the reporter and conditional allele [34].

### 2.2. Cancer

Neumann et al. have generated the first *Prdx1*-knockout mice (*Prdx1*^−/−^) [15]. The knockout mouse strain targets exon 3 of the *Prdx1* gene by the insertion of a transposon containing a PGK-neomycin-resistant (neo^R^) cassette, which disrupts all protein-coding transcripts [15]. The knockout mice are viable and fertile but show severe hemolytic anemia and several malignant cancers, including lymphomas, sarcomas, and carcinomas, which cause a shortened life span [15]. Using the same knockout mice, Neumann’s group indicated that PRDX1 is involved in Pten-mediated tumor suppression in Ras-induced breast cancer [16] and inhibition of fibroblast transition into cancer-associated fibroblasts (CAFs) [18]. The tumor suppressor function of PRDX1 was also demonstrated in another study using a different *Prdx1*-knockout mouse strain, which was generated using the Lexicon gene trap ES cell clone, which has a gene trap vector in intron 3 of the *Prdx1* gene [17]. Elevated nuclear ROS on primary tissues isolated from the *Prdx1*^−/−^ leads to increased DNA damage and tumor susceptibility [17].

### 2.3. Erythrocytes

Severe hemolytic anemia with defects in blood parameters, which is observed in Neumann’s *Prdx1*-knockout mice, is not observed in other *Prdx1*-knockout mouse models [32,33], although *Prdx1* deficiency aggravates hemolytic anemia symptoms in *Prdx2*-knockout mice [32]. These knockout mouse strains have been generated by replacing whole exons of the *Prdx1* gene with a neo^R^ cassette [32] or using the gene trap clone [33].

### 2.4. Inflammation

In different inflammation disease models, *Prdx1*^−/−^ show conflicting results. PRDX1 enhances cerebral ischemia–reperfusion (I/R) injuries by activation of inflammation and apoptosis [19], and it initiates inflammation in the ozone-exposed lung [20]. *Prdx1* deficiency, however, aggravates pulmonary inflammation and fibrosis in the bleomycin-treated model [26]. Atherosclerosis and chronic inflammation model mice (*Prdx1*^−/−^;*ApoE*^−/−^) show increased formation of atherosclerotic plaque compared with *Prdx1*^+/+^;*ApoE*^−/−^ mice [13,21]. *Prdx1*-deficient macrophages present impaired lipophagic flux and cholesterol homeostasis [13]. Lipopolysaccharide (LPS)-induced lung injury, lethal shock, and neuroinflammation are increased in *Prdx1*^−/−^ [22,23,24]. PRDX1 is a negative regulator of Th2-type allergic asthma that is induced by ovalbumin [25]. Inflammatory stimuli produce the intramolecular disulfide bond in HMGB1, which is mediated by PRDX1 or PRDX2 [35]. The formation of the disulfide bond is sufficient for HMGB1 secretion and secreted HMGB1 signals danger to surrounding cells. HMGB1 secretion induced by LPS is attenuated in macrophages isolated from *Prdx1*- or *Prdx2*-knockout mice [35].

### 2.5. Others

PRDX1 interacts with Gde2 and reduces the formation of an intramolecular disulfide bond between the N- and C-terminal regions of Gde2, which induces neuronal differentiation [36]. *Prdx1* deficiency attenuates cisplatin-induced nephrotoxicity [37]. Studies using *Prdx1*-knockout mouse models also show that PRDX1 is involved in maintenance of stemness of mouse embryonic stem cells by suppression of ROS/JNK-induced neurogenesis [38], modulation of cellular senescence in mouse embryonic fibroblasts (MEFs) [39], host defenses against *Mycobacterium tuberculosis* and *Staphylococcus aureus* [40,41], and maintenance of progesterone production in the corpus luteum through regulating the unfolded protein response [42].

## 3. PRDX2

### 3.1. Genetics and Knockout Mouse Strains

PRDX2 is a cytosolic typical 2-Cys PRDX and has a similar structure to that of PRDX1 [7]. Mouse PRDX1 and PRDX2 proteins share 89% sequence similarity and 74% sequence identity and perform overlapping and distinct biological functions [31]. The *Prdx2* gene is on mouse chromosome 8 and there are five alternative forms of *Prdx2* protein-coding transcripts [31]. The first *Prdx2*-knockout mice (*Prdx2*^−/−^) have been generated by replacing the genomic DNA encoding exons 1-5 with a neo^R^ cassette. They are viable and fertile [43] and most studies have used the same *Prdx2*-knockout mouse model. ES cell clones possessing the reporter and conditional allele of the *Prdx2* gene have been produced [34], but a *Prdx2*-knockout mouse strain with conditional potential has not been reported.

### 3.2. Erythrocytes

Typical phenotypes of *Prdx2*^−/−^ are hemolytic anemia, splenomegaly, Heinz body formation, and morphologically abnormal red blood cells [43]. PRDX2 is expressed in all cell types and is the third most abundant protein in erythrocytes [44]. Three PRDX isoforms (PRDX1, PRDX2, and PRDX6) are expressed in mature erythrocytes and PRDX2 is the most abundant protein among them [45]. The role of PRDX2 in protecting erythrocytes against oxidative stress has been verified by several studies using the same *Prdx2*-knockout mouse model [46,47,48,49,50,51,52,53,54,55]. PRDX2 has roles to protect erythrocytes from ROS-mediated DNA damage during erythropoiesis [48] and to protect hemoglobin from oxidative stress [49,50]. The decameric structure of PRDX2 binds to hemoglobin to stabilize and protect the protein [49]. Studies using *Prdx2*^−/−^ show that PRDX2 is involved in the homeostasis of iron and membrane proteins of erythrocytes, as well as cellular senescence of erythrocytes and skin cells [51,53,56]. Erythrocytes lose PRDX2 protein gradually during the life span of erythrocytes [54]. PRDX2 is hyperoxidized by H_2_O_2_ and the hyperoxidized PRDX2 is degraded by the 20S proteasome [54].

### 3.3. Blood Vessels

PRDX2 is also involved in the homeostasis of blood vessels [57,58,59]. The redox-sensitive transcription factor Nrf2 activates PRDX2 expression to protect vascular smooth muscle cells from oxidative vascular injury [57]. In vascular endothelial cells, VEGFR2 no longer responds to VEGF stimulation by the oxidative-stress-induced formation of a cysteine disulfide bond in the C-terminal region of VEGFR2 [58]. PRDX2, not PRDX1, inhibits the formation of the intramolecular disulfide bond in VEGFR2 [58]. Furthermore, tumor angiogenesis is suppressed in *Prdx2*^−/−^ [58]. The antioxidant activity of PRDX2 needs negative regulation of collagen-stimulated platelet activation and platelet-dependent thrombosis [60]. Among 2-Cys Prdxs, *Prdx2* deficiency exacerbates the neointimal hyperplasia induced by the balloon injury of the carotid arteries [59].

### 3.4. Immune Responses

ROS are harmful byproducts but are also essential for immune responses [61] and their scavenger, PRDX2, inhibits immune cell responsiveness [62,63]. Increased exposure to ROS by *Prdx2* deficiency activates the proliferation of T lymphocytes and the differentiation of dendritic cells [62,64]. Like *Prdx1*^−/−^, *Prdx2*^−/−^ are sensitive to LPS-induced inflammatory responses, including lethal shock [65]. LPS activates inflammatory responses which are mediated by NADPH-oxidase-derived ROS generation in *Prdx2*-deficient macrophages [65]. *Prdx2* deficiency increases immune cell accumulation in atherosclerotic lesions, which exacerbates atherosclerosis in *ApoE*^−/−^ mice [66]. Hypoxia-induced oxidative stress in the lung of *Prdx2*^−/−^ causes an amplified inflammatory response, vascular dysfunction, and autophagy activation, which lead to the development of pulmonary arterial hypertension [67]. *Prdx2* deficiency ameliorates dextran sulfate sodium (DSS)-induced colitis by enhancing the development of Foxp3^+^ regulatory T cells [68].

### 3.5. Cancer

In the *Apc*^+/Min^ colorectal cancer model, the depletion of *Prdx2* inactivates the formation of intestinal adenomatous polyposis through Axin/β-catenin signaling [69,70]. Increased intracellular H_2_O_2_ level by the *Apc* mutation leads to the direct binding of PRDX2 to a poly(ADP-ribose) polymerase (PARP) tankyrase. This binding protects the tankyrase from its oxidative inactivation, and thus induces PARP-dependent Axin degradation [69,70].

### 3.6. Bone

*Prdx2*^−/−^ have higher levels of bone mass than those of wild-type mice because PRDX2 is a negative regulator of BMP2-induced osteoblast differentiation [45]. PRDX2 also functions as a negative regulator of LPS-induced osteoclastogenesis and bone loss, which are induced by ROS-mediated JNK and STAT3 activation [71].

### 3.7. Others

PRDX2 is important for homeostasis of other tissues. PRDX2 protects hippocampal neurons from age-dependent mitochondrial decay [72] and maintains the stemness of mouse embryonic stem cells [38]. Oxidation of protein tyrosine phosphatases by ROS in *Prdx2*^−/−^ fed a high-fat diet causes reduced body weight and increased glucose clearance [73]. PRDX2 controls corpus luteum regression that is induced by prostaglandin F2α-mediated ROS and protects against age-related ovarian failure [74,75].

## 4. Prdx3

### 4.1. Genetics and Knockout Mouse Strains

PRDX3 is a member of typical 2-Cys PRDXs and is mainly localized in mitochondria due to a mitochondrial targeting sequence at the N-terminal region. The *Prdx3* gene is localized on mouse chromosome 19 and there is a *Prdx2* protein-coding transcript [31]. Two knockout mouse lines have been analyzed to study the in vivo function of PRDX3. The first knockout mouse line was produced in 2007 using an ES cell clone generated by the gene trap approach [76]. In this ES clone, the VICTR20 gene trap vector [77] is inserted in intron 1 of the *Prdx3* gene [76]. The second knockout mouse line was generated by the homologous recombination approach [78]. The genomic DNA region possessing exons 1-4 of the *Prdx3* gene is replaced with the neo^R^ cassette in the knockout mouse line [78]. ES cell clones possessing the reporter and conditional allele of the *Prdx3* gene have been produced [34], but no *Prdx3*-knockout mouse strain with conditional potential has been reported.

### 4.2. Muscles

PRDX3 is important to protect mitochondria against oxidative stress. *Prdx3* deficiency leads to reduced mitochondrial DNA content and ATP production and impaired mitochondrial fusion [79,80]. Mitochondrial homeostasis is necessary for the proper function of skeletal muscles. PRDX3 has roles in the prolonged contraction of skeletal muscles and physical strength [79,80].

### 4.3. Metabolism

PRDX3 is involved in metabolic homeostasis. *Prdx3*^−/−^ show increased fat mass by adipocyte hypertrophy, impaired mitochondrial enzymes, and adipokine dysregulation, resulting in impaired glucose tolerance and insulin resistance [78].

### 4.4. Others

PRDX3 protects the lungs from LPS-induced damages, such as 8-hydroxy-2′-deoxyguanosine (8-OHdG) formation and protein carbonylation [76]. PRDX3 also protects macrophages and the liver against LPS-induced oxidative stress and pyrazole-induced oxidative damage, respectively [81,82]. Increased oxidative stress in *Prdx3*^−/−^ shows placental defects, including focal necrosis and hyaline degeneration in trophoblast giant cells and vessel degeneration [83,84]. PRDX3 also has a protective role in UV-induced apoptosis of epidermal keratinocytes [85].

## 5. Prdx4

### 5.1. Genetics and Knockout Mouse Strains

PRDX4 is a member of typical 2-Cys PRDXs. *Prdx4* is on the X chromosome and produces two forms of alternative transcripts [31]. Each of them uses a different exon 1 (exon 1A and exon 1B) [86]. All tissues, including the testis, express *Prdx4* mRNAs transcribed from exon 1B, which encodes the cleavable N-terminal signal sequence, whereas the testis produces a testis-specific form of *Prdx4* mRNAs transcribed from exon 1A [86]. PRDX4 is predominantly present in the ER and secreted to extracellular space [87,88]. A *Prdx4*-knockout mouse strain has been widely used to study in vivo functions of PRDX4 [89]. Originally, the knockout mouse line was generated with conditional potential by insertion of two *loxP* sequences flanking exon 1B of the *Prdx4* gene [89]. However, most studies have used *Prdx4*-null mice, in which exon 1B is deleted. The testis-specific form of PRDX4 is not deleted in this knockout mouse strain [86].

### 5.2. Phenotypes

*Prdx4*-knockout male mice (*Prdx4*^−/y^) are fertile but show testicular atrophy [89]. Spermatogenic cells in *Prdx4*^−/y^ are susceptible to cell death by oxidative stress [89]. In a DSS-induced colitis model, *Prdx4*^−/y^ show loss of body weight and shortening of colon length, which may be caused by ER stress and oxidative damage in colonic epithelial cells [90]. *Prdx4*^−/y^ show a higher incidence of hepatocellular carcinoma in the diethylnitrosamine injection model compared with that of wild-type mice [91]. Triple deletion of ER thiol oxidases, *Ero1l* and *Ero1lb*, and *Prdx4* causes interfered procollagen maturation and thus forms defective connective tissues in the extracellular matrix [92]. *Prdx4* and superoxide dismutase 1 (*Sod1*) double-knockout mice (*Prdx4*^−/y^;*Sod*^−/−^) show more severe liver phenotypes, such as aggravated liver steatosis and liver failure, at a relatively young age compared with those of wild-type, *Prdx4*^−/y^, and *Sod*^−/−^ [93].

## 6. Prdx5

### 6.1. Genetics and Knockout Mouse Strains

PRDX5 is a unique member of the atypical 2-Cys subfamily in mammals and is ubiquitously expressed in tissues [5,12]. PRDX5 is present in the cytosol, peroxisomes, and mitochondria [8]. The *Prdx5* gene is located on mouse chromosome 19 and there are four alternative forms of *Prdx5* protein-coding transcripts [31]. A *Prdx5*-knockout mouse line has been generated by the homologous recombination approach [94]. Another *Prdx5*-knockout mouse line that is generated by the gene trap approach is commercially available [95]. In this knockout line, a gene trap vector is inserted in the 5′UTR region of the *Prdx5* gene. ES cell clones possessing the reporter and conditional allele of the *Prdx5* gene have been produced [34], but a *Prdx5*-knockout mouse strain with conditional potential has not been reported.

### 6.2. Metabolism

*Prdx5* deficiency leads to increased susceptibility to high-fat-diet-induced obesity, and thus *Prdx5*-knockout mice fed a high-fat diet show several metabolic abnormalities, including increased body weight, adipocyte hypertrophy, fat accumulation in the liver, hepatic steatosis, and an increased triglyceride level in the serum [94,96].

## 7. Prdx6

### 7.1. Genetics and Knockout Mouse Strains

In mammals, PRDX6 is a unique member of the 1-Cys subfamily [5,12]. The *Prdx6* gene is located on mouse chromosome 1 and produces two forms of alternatively spliced protein-coding transcripts [31]. PRDX6 is widely expressed in tissues and localized in the cytosol [8,97]. Two *Prdx6*-knockout mouse lines have been generated [97,98]. Exons 1 and 2 of the *Prdx6* gene are replaced by a LacZ reporter and a neo^R^ cassette in the first knockout mouse line [97], and a part of exon 3 is replaced by a neo^R^ cassette in the second line [98]. Both knockout mouse lines are viable, fertile, and display no gross morphological defects [97,98]. IMPC produced a *Prdx6*-conditional knockout mouse line [34], although the mouse line has not been used for detailed phenotyping.

### 7.2. Tissue Protection

The protective roles of PRDX6 in the lung have been analyzed with *Prdx6*^−/−^. Administration of paraquat, an herbicide that produces damaging ROS within cells, causes tissue damage, decreased survival rate, and increased oxidation of lipids and proteins in the lungs of *Prdx6*^−/−^ [97,99]. Exposure to 100% oxygen leads to similar defects in the lungs of *Prdx6*^−/−^ [100]. Comparison between glutathione peroxidase 1 (Gpx1)-knockout mice and *Prdx6*^−/−^ reveals that PRDX6 is the major enzyme for the reduction of phospholipid hydroperoxides in the lung [101]. In addition to the glutathione-dependent peroxidase activity, PRDX6 also has phospholipaseA2 (PLA_2_) and lysophospholipid:acyltransferase activities [102,103]. The deficiency of PLA_2_ activity alters phospholipid metabolism in the lungs of *Prdx6*^−/−^ [102]. The lung and pulmonary microvascular endothelial cells (PMVECs) isolated from *Prdx6*^−/−^ show increased sensitivity to peroxidative stress induced by exposure to 100% oxygen or *tert*-butyl hydroperoxide (t-BOOH) treatment [104,105]. These defects are partially rescued by the expression of mutant PRDX6 with either peroxidase activity alone or PLA_2_ activity alone [104,105]. However, coexpression of these mutant forms of PRDX6 rescues *Prdx6*-null PMVECs treated with t-BOOH as well as the expression of wild-type PRDX6 [104]. The glutathione-dependent peroxidase activity of PRDX6 can reduce both short-chain hydroperoxides such as H_2_O_2_ and phospholipid hydroperoxides [106]. The repair of peroxidized cell membranes of the lung or PMVECs is mostly dependent on the phospholipid hydroperoxidase activity rather than peroxidase activity toward H_2_O_2_ [106]. Protective effects of PRDX6 in the lung have been revealed using other lung injury models, including exposure to H_2_O_2_, LPS, or chronic cigarette smoke and cecal ligation and puncture (CLP)-induced acute lung injury [107,108,109,110]. Interestingly, the treatment of angiotensin II or phorbol ester increases the generation of superoxide and H_2_O_2_ in wild-type PMVECs but not in *Prdx6*-deficient PMVECs [111,112]. The authors explain that the PLA_2_ activity of PRDX6 is necessary for the activation of NADPH oxidase type 2 (NOX2), which produces superoxide [111,112]. PRDX6 also has protective roles in other tissues. PRDX6 protects the kidney from metabolic acidosis by contributing to the maintenance of anion exchanger 1 [113] and blood vessels in wounded skin [114].

### 7.3. Prion Disease

In ME7-infected prion disease models, *Prdx6* deficiency worsens prion-related neuropathology [115]. These defects are caused by *Prdx6* deficiency in astrocytes because PRDX6 is predominantly expressed in astrocytes rather than neurons in the brain [115].

### 7.4. Inflammation and Metabolism

Oxidative stress contributes to the pathogenesis of various inflammatory and metabolic diseases. Hepatic I/R injury causes a significant increase of PRDX6 expression and PRDX6 transfer from the cytoplasm to the mitochondria [116]. *Prdx6* deficiency in the I/R model increases the mitochondrial generation of H_2_O_2_ and mitochondrial dysfunction, thus leading to severe hepatocellular damage [116]. In *Prdx6*^−/−^, ethanol-induced lipid accumulation and peroxidation are observed in the liver [117]. An intensive study shows that *Prdx6*^−/−^ develop insulin resistance, diabetic dyslipidemia, impaired insulin signaling, morphological changes in the pancreas and liver, and increased pro-inflammatory responses, suggesting that *Prdx6* deficiency is a key mediator of hyperglycemia in type 2 diabetes [118]. A study suggests that PRDX6 is involved in the biosynthesis of fatty acid esters of hydroxy fatty acids that are lipid mediators with potent antidiabetic and anti-inflammatory activities [119]. In acute and chronic DSS-induced colitis models, however, *Prdx6* deficiency attenuates the development of colitis [120]. The authors explain that *Prdx6* deficiency is compensated by the upregulation of other PRDXs (PRDX3 and PRDX4) and antioxidant enzymes (Nrf2, Gss, and Gclm). A study tested the relationship between PRDX6 and atherosclerosis using *Prdx6*^−/−^ with three different genetic backgrounds: atherosclerosis-resistant 129/SvJ (129), atherosclerosis-susceptible B6, and mixed B6;129 [121]. The effects of *Prdx6* deficiency in atherosclerosis are minor and background dependent.

### 7.5. Aging

*Prdx6*^−/−^ show age-related phenotypes [122,123,124]. *Prdx6* deficiency decreases the fertility of male *Prdx6*^−/−^ in an age-dependent manner [122]. PRDX6 protects spermatozoa against the oxidative stress that causes protein oxidation, lipid peroxidation, and DNA oxidation and fragmentation [122,123]. Lens epithelial cells (LECs) isolated from *Prdx6*^−/−^ display elevated ROS expression and ER-stress-associated phenotypes [124,125]. Human LECs derived from aged men show a decreased level of PRDX6 and ER-stress-associated phenotypes [124]. LECs of *Prdx6*^−/−^ are also more vulnerable to UV irradiation than those of wild-type mice [126].

### 7.6. Cancer

*Prdx6* deficiency enhances susceptibility to tumorigenesis in the human-papillomavirus-8-induced skin cancer model [127]. The anti-tumorigenic effect of PRDX6 is achieved by the reduction of oxidative stress rather than altered proliferation, apoptosis, or the inflammatory response in keratinocytes [127].

## 8. Conclusions

PRDXs are typical peroxidases for the removal of cellular peroxides [1,2]. To investigate the biological roles of PRDXs, numerous approaches have been performed using the cell culture system. Although these experiments have provided valuable insights into PRDX biology, the approaches are not sufficient to reveal physiological functions in the human body. Knockout mouse models offer more reliable data to understand the in vivo functions of PRDXs (Table 1). More than one knockout mouse model of each PRDX has been generated and analyzed intensively. These knockout mouse models show that each PRDX functions essentially as a similar peroxidase and also performs specific functions depending on organs or intracellular organelles. In the past, the generation of knockout mouse models was time-consuming and labor-intensive work. However, recently developed gene-editing techniques using the CRISPR/Cas9 system have dramatically reduced these efforts [128]. Now, we can easily obtain GEM models that are more precisely modified than the previous complete knockout mouse models. Future studies using these new models, as well as complete knockout mouse models, will help us better understand the physiological roles of PRDXs and provide possible therapeutic targets for drugs against diseases, such as cancer and inflammatory and metabolic diseases.

## Figures and Tables

**Table 1:** Knockout mouse models of Prdxs.

Gene	Models	Phenotypes	Challenges ^1^	Affected Organs/Cells
*Prdx1*	KO1 [15] (Homologous recombination, Insertion, exon 3) ^2^	Tumorigenesis [15,16,18]	None	Various
		Hemolytic anemia [15]	None	Red blood cells (RBCs)
		Atherosclerosis [21]	Normal diet, *ApoE*^−/−^	Aorta
		Neuronal defects [36]	None	Embryonic neurons
	KO2 [32] (Homologous recombination, Replacement, exons 1–6)	Pro-inflammation [23]	Lipopolysaccharide (LPS)	Liver
		Defective RBC clearance [32]	None	Macrophages
		Cellular senescence [39]	None	Mouse embryonic fibroblasts (MEFs)
		Defective host defense [41]	*Staphylococcus aureus*	Liver, lung
	KO3 [17] (Gene Trap, Insertion, Intron 3)	Tumorigenesis [17]	None	Brain, liver, spleen, MEFs
	KO4 [33] (Gene Trap, Insertion, Intron 3)	Anti-inflammation [20]	Ozone	Lung
		Pro-inflammation [26]	Bleomycin	Lung
		Fibrosis [26]	Bleomycin	Lung
		Asthma [25]	Cisplatin	Lung
		Defective host defense [40]	*Mycobacterium tuberculosis*	Lung
		Kidney defects [37]	Ovalbumin	Kidney
		Pro-apoptosis [28]	UVA	MEFs
	Uncertain	Anti-inflammation [19]	Ischemia–reperfusion (I/R) injury	Brain
		Pro-inflammation [24]	LPS	Microglia
		Anti-apoptosis [19]	I/R injury	Brain
		Pro-apoptosis [42]	Tunicamycin	Corpus luteum
		Atherosclerosis [13]	Normal or high-fat diet, *ApoE*^−/−^	Aorta
		Lung defects [22]	LPS	Lung, macrophages
		Loss of stemness [38]	Differentiation medium	Embryonic stem (ES) cells
		Reduced progesterone [42]	Tunicamycin	Corpus luteum
		Defective HMGB1 secretion [35]	LPS	Macrophages
*Prdx2*	KO1 [43] (Homologous recombination, Replacement, exons 1–5)	Hemolytic anemia [32,43,46,47,48,49,50,51,52,53,54,55]	None	RBCs, spleen, bone marrow (BM)
		Defective iron homeostasis [53]	Carbonyl-iron, LPS	RBCs, liver, BM
		Blood vessel defects [58,59]	None	Endothelial cells
			Balloon injury	Carotid arteries
		Platelet defects [60]	Collagen	Platelet
		Pro-inflammation [62,63,64,65]	None	Spleen, BM, thymus
			LPS	Macrophages
		Anti-inflammation [68]	Dextran sulfate sodium (DSS)	Colon
		Atherosclerosis [66]	Atherogenic cholate-containing diet, *ApoE*^−/−^	Aorta
		Anti-tumorigenesis [69,70]	*Apc* ^+/Min^	Intestine, colon
		Metabolic defects [73]	High-fat diet	MEFs, serum, muscle
		Ovary defects [74,75]	None, PGF2α, 4-vinylcyclohexene diepoxide	Ovary
		Bone defects [45,71]	LPS	Bone
		Neuronal defects [72]	None	Neurons
		Pulmonary hypertension [67]	Hypoxia	Lung
		Cellular senescence [56]	None	MEFs
		Loss of stemness [38]	Differentiation medium	ES cells
		Defective HMGB1 secretion [35]	LPS	Macrophages
	Uncertain	Blood vessel defects [57]	FeCl_3_	Carotid arteries
*Prdx3*	KO1 [78] (Homologous recombination, Replacement, exons 1–4)	Pro-apoptosis [85]	UVB	Keratinocytes
		Muscle defects [79]	None	Muscle
		Liver defects [82]	Pyrazole	Liver
		Metabolic defects [78]	None	Fat, adipocytes
	KO2 [76] (Gene Trap, Insertion, Intron 1)	Pro-apoptosis [80]	None	Brain
		Muscle defects [80]	None	Muscle
		Lung defects [76]	LPS	Lung
		Macrophage defects [81]	LPS	Macrophages
		Placental defects [83,84]	None	Placenta
*Prdx4*	KO1 [89] (Homologous recombination, Cre-*loxP* deletion, exon 1)	Defective spermatogenesis [89]	None	Testis
		Tumorigenesis [91]	Diethylnitrosamine	Liver
		Colon defects [90]	Dextran sulfate sodium	Colon
		Liver defects [93]	*Sod1* ^−/−^	Liver
		Defective connective tissues [92]	*Ero1l*^−/−^, *Ero1bl*^−/−^	Connective tissues
*Prdx5*	KO1 [94] (Homologous recombination, Replacement, not specified)	Metabolic defects [94,96]	High-fat diet	Fat, liver
*Prdx6*	KO1 [97] (Homologous recombination, Replacement, exons 1 and 2)	Tissue defects [97]	Paraquat	Lung, kidney, liver, macrophages
		Anti-inflammation [120]	DSS	Colon
		Tumorigenesis [127]	Human papillomavirus 8	Skin
		Atherosclerosis (mild) [121]	Atherogenic high fat diet	Aorta, plasma
		Metabolic defects [118]	None	Various
		Lens defects [124,125]	None, hypoxia, CoCl_2_, tunicamycin, H_2_O_2_,	Lens epithelial cells (LECs)
		Lens defects and pro-apoptosis [126]	UVB	LECs
		Liver defects [117]	Ethanol	Liver
		Prion disease [115]	ME7	Brain
		Vascular defects [114]	UV	Skin, blood vessels
	KO2 [98] (Homologous recombination, Replacement, exon 3)	Lung defects [99,100,101,107,108,109,110]	Paraquat	Lung
			Hyperoxia	Lung
			Hyperoxia, *tert*-butylhydroperoxide (t-BOOH), Paraquat	Lung, pulmonary microvascular endothelial cells (PMVECs)
			H_2_O_2_	Type II alveolar epithelial cells
			Cigarette smoke	Lung
			Cecal ligation and puncture	Lung
			LPS	Lung
		Lung defects (phospholipid metabolism) [102]	None	Lung
		PMVEC defects [104]	t-BOOH	PMVECs
		Lung and PMVEC defects [105,106]	t-BOOH, hyperoxia	PMVECs, lung
		Attenuated production of superoxide and H_2_O_2_ [111,112]	Angiotensin II, Phorbol ester	Lung, PMVECs, alveolar macrophages
		Defective spermatogenesis [122,123]	Aging, t-BOOH	Sperm
		Liver defects [116]	I/R injury	Liver
		Kidney defects [113]	NH_4_Cl	Kidney

^1^ Most phenotypes are induced by various challenges. ^2^ Methods to generate knockout mouse models.

## References

[1] Rhee S.G. (2016). Overview on Peroxiredoxin. Mol. Cells.

[2] Kim Y., Jang H.H. (2019). The Role of Peroxiredoxin Family in Cancer Signaling. J. Cancer Prev..

[3] Nelson K.J., Knutson S.T., Soito L., Klomsiri C., Poole L.B., Fetrow J.S. (2011). Analysis of the peroxiredoxin family: Using active-site structure and sequence information for global classification and residue analysis. Proteins.

[4] Chae H.Z., Robison K., Poole L.B., Church G., Storz G., Rhee S.G. (1994). Cloning and sequencing of thiol-specific antioxidant from mammalian brain: Alkyl hydroperoxide reductase and thiol-specific antioxidant define a large family of antioxidant enzymes. Proc. Natl. Acad. Sci. USA.

[5] Rhee S.G., Kang S.W., Chang T.S., Jeong W., Kim K. (2001). Peroxiredoxin, a novel family of peroxidases. IUBMB Life.

[6] Wood Z.A., Schroder E., Robin Harris J., Poole L.B. (2003). Structure, mechanism and regulation of peroxiredoxins. Trends Biochem. Sci..

[7] Hall A., Karplus P.A., Poole L.B. (2009). Typical 2-Cys peroxiredoxins–structures, mechanisms and functions. FEBS J..

[8] Rhee S.G., Woo H.A., Kil I.S., Bae S.H. (2012). Peroxiredoxin functions as a peroxidase and a regulator and sensor of local peroxides. J. Biol. Chem..

[9] Seo M.S., Kang S.W., Kim K., Baines I.C., Lee T.H., Rhee S.G. (2000). Identification of a new type of mammalian peroxiredoxin that forms an intramolecular disulfide as a reaction intermediate. J. Biol. Chem..

[10] Hall A., Nelson K., Poole L.B., Karplus P.A. (2011). Structure-based insights into the catalytic power and conformational dexterity of peroxiredoxins. Antioxid. Redox Signal..

[11] Soito L., Williamson C., Knutson S.T., Fetrow J.S., Poole L.B., Nelson K.J. (2011). PREX: PeroxiRedoxin classification indEX, a database of subfamily assignments across the diverse peroxiredoxin family. Nucleic Acids Res..

[12] PeroxiRedoxin Classification IndEX. http://csb.wfu.edu/prex.test/.

[13] Jeong S.J., Kim S., Park J.G., Jung I.H., Lee M.N., Jeon S., Kweon H.Y., Yu D.Y., Lee S.H., Jang Y. (2018). Prdx1 (peroxiredoxin 1) deficiency reduces cholesterol efflux via impaired macrophage lipophagic flux. Autophagy.

[14] Mouse Genome Informatics. http://www.informatics.jax.org/.

[15] Neumann C.A., Krause D.S., Carman C.V., Das S., Dubey D.P., Abraham J.L., Bronson R.T., Fujiwara Y., Orkin S.H., Van Etten R.A. (2003). Essential role for the peroxiredoxin Prdx1 in erythrocyte antioxidant defence and tumour suppression. Nature.

[16] Cao J., Schulte J., Knight A., Leslie N.R., Zagozdzon A., Bronson R., Manevich Y., Beeson C., Neumann C.A. (2009). Prdx1 inhibits tumorigenesis via regulating PTEN/AKT activity. EMBO J..

[17] Egler R.A., Fernandes E., Rothermund K., Sereika S., de Souza-Pinto N., Jaruga P., Dizdaroglu M., Prochownik E.V. (2005). Regulation of reactive oxygen species, DNA damage, and c-Myc function by peroxiredoxin 1. Oncogene.

[18] Jezierska-Drutel A., Attaran S., Hopkins B.L., Skoko J.J., Rosenzweig S.A., Neumann C.A. (2019). The peroxidase PRDX1 inhibits the activated phenotype in mammary fibroblasts through regulating c-Jun N-terminal kinases. BMC Cancer.

[19] Liu Q., Zhang Y. (2019). PRDX1 enhances cerebral ischemia-reperfusion injury through activation of TLR4-regulated inflammation and apoptosis. Biochem. Biophys. Res. Commun..

[20] Yanagisawa R., Warabi E., Inoue K., Yanagawa T., Koike E., Ichinose T., Takano H., Ishii T. (2012). Peroxiredoxin I null mice exhibits reduced acute lung inflammation following ozone exposure. J. Biochem..

[21] Kisucka J., Chauhan A.K., Patten I.S., Yesilaltay A., Neumann C., Van Etten R.A., Krieger M., Wagner D.D. (2008). Peroxiredoxin1 prevents excessive endothelial activation and early atherosclerosis. Circ. Res..

[22] Lv W.P., Li M.X., Wang L. (2017). Peroxiredoxin 1 inhibits lipopolysaccharide-induced oxidative stress in lung tissue by regulating P38/JNK signaling pathway. Eur. Rev. Med. Pharmacol. Sci..

[23] Sun H.N., Feng L., Wang A.G., Wang J.Y., Liu L., Jin M.H., Shen G.N., Jin C.H., Lee D.S., Kwon T.H. (2018). Peroxiredoxin I deficiency increases LPSinduced lethal shock in mice. Mol. Med. Rep..

[24] Kim S.U., Park Y.H., Min J.S., Sun H.N., Han Y.H., Hua J.M., Lee T.H., Lee S.R., Chang K.T., Kang S.W. (2013). Peroxiredoxin I is a ROS/p38 MAPK-dependent inducible antioxidant that regulates NF-kappaB-mediated iNOS induction and microglial activation. J. Neuroimmunol..

[25] Inoue K., Takano H., Koike E., Warabi E., Yanagawa T., Yanagisawa R., Ishii T. (2009). Peroxiredoxin I is a negative regulator of Th2-dominant allergic asthma. Int. Immunopharmacol..

[26] Kikuchi N., Ishii Y., Morishima Y., Yageta Y., Haraguchi N., Yamadori T., Masuko H., Sakamoto T., Yanagawa T., Warabi E. (2011). Aggravation of bleomycin-induced pulmonary inflammation and fibrosis in mice lacking peroxiredoxin I. Am. J. Respir. Cell Mol. Biol..

[27] Guo W., Liu X., Li J., Shen Y., Zhou Z., Wang M., Xie Y., Feng X., Wang L., Wu X. (2018). Prdx1 alleviates cardiomyocyte apoptosis through ROS-activated MAPK pathway during myocardial ischemia/reperfusion injury. Int. J. Biol. Macromol..

[28] Ito T., Kimura S., Seto K., Warabi E., Kawachi Y., Shoda J., Tabuchi K., Yamagata K., Hasegawa S., Bukawa H. (2014). Peroxiredoxin I plays a protective role against UVA irradiation through reduction of oxidative stress. J. Dermatol. Sci..

[29] Jang H.H., Kim S.Y., Park S.K., Jeon H.S., Lee Y.M., Jung J.H., Lee S.Y., Chae H.B., Jung Y.J., Lee K.O. (2006). Phosphorylation and concomitant structural changes in human 2-Cys peroxiredoxin isotype I differentially regulate its peroxidase and molecular chaperone functions. FEBS Lett..

[30] Nassour H., Wang Z., Saad A., Papaluca A., Brosseau N., Affar el B., Alaoui-Jamali M.A., Ramotar D. (2016). Peroxiredoxin 1 interacts with and blocks the redox factor APE1 from activating interleukin-8 expression. Sci. Rep..

[31] Cunningham F., Achuthan P., Akanni W., Allen J., Amode M.R., Armean I.M., Bennett R., Bhai J., Billis K., Boddu S. (2019). Ensembl 2019. Nucleic Acids Res..

[32] Han Y.H., Kwon T., Kim S.U., Ha H.L., Lee T.H., Kim J.M., Jo E.K., Kim B.Y., Yoon D.Y., Yu D.Y. (2012). Peroxiredoxin I deficiency attenuates phagocytic capacity of macrophage in clearance of the red blood cells damaged by oxidative stress. BMB Rep..

[33] Uwayama J., Hirayama A., Yanagawa T., Warabi E., Sugimoto R., Itoh K., Yamamoto M., Yoshida H., Koyama A., Ishii T. (2006). Tissue Prx I in the protection against Fe-NTA and the reduction of nitroxyl radicals. Biochem. Biophys. Res. Commun..

[34] Dickinson M.E., Flenniken A.M., Ji X., Teboul L., Wong M.D., White J.K., Meehan T.F., Weninger W.J., Westerberg H., Adissu H. (2016). High-throughput discovery of novel developmental phenotypes. Nature.

[35] Kwak M.S., Kim H.S., Lkhamsuren K., Kim Y.H., Han M.G., Shin J.M., Park I.H., Rhee W.J., Lee S.K., Rhee S.G. (2019). Peroxiredoxin-mediated disulfide bond formation is required for nucleocytoplasmic translocation and secretion of HMGB1 in response to inflammatory stimuli. Redox Biol..

[36] Yan Y., Sabharwal P., Rao M., Sockanathan S. (2009). The antioxidant enzyme Prdx1 controls neuronal differentiation by thiol-redox-dependent activation of GDE2. Cell.

[37] Okada K., Ma D., Warabi E., Morito N., Akiyama K., Murata Y., Yamagata K., Bukawa H., Shoda J., Ishii T. (2013). Amelioration of cisplatin-induced nephrotoxicity in peroxiredoxin I-deficient mice. Cancer Chemother. Pharmacol..

[38] Kim S.U., Park Y.H., Kim J.M., Sun H.N., Song I.S., Huang S.M., Lee S.H., Chae J.I., Hong S., Sik Choi S. (2014). Dominant role of peroxiredoxin/JNK axis in stemness regulation during neurogenesis from embryonic stem cells. Stem Cells.

[39] Park Y.H., Kim H.S., Lee J.H., Choi S.A., Kim J.M., Oh G.T., Kang S.W., Kim S.U., Yu D.Y. (2017). Peroxiredoxin I participates in the protection of reactive oxygen species-mediated cellular senescence. BMB Rep..

[40] Matsumura K., Iwai H., Kato-Miyazawa M., Kirikae F., Zhao J., Yanagawa T., Ishii T., Miyoshi-Akiyama T., Funatogawa K., Kirikae T. (2016). Peroxiredoxin 1 Contributes to Host Defenses against Mycobacterium tuberculosis. J. Immunol..

[41] Sun H.N., Liu Y., Wang J.N., Wang C., Liu R., Kong L.Z., Zhen X., Chandimali N., Cui Y.D., Kim S.U. (2019). Protective Role of Peroxiredoxin I in Heat-Killed Staphylococcus Aureus-infected Mice. In Vivo.

[42] Park H.J., Lee D.G., Seong J.B., Lee H.S., Kwon O.S., Kang B.S., Park J.W., Lee S.R., Lee D.S. (2018). Peroxiredoxin I maintains luteal function by regulating unfolded protein response. Reprod. Biol. Endocrinol..

[43] Lee T.H., Kim S.U., Yu S.L., Kim S.H., Park D.S., Moon H.B., Dho S.H., Kwon K.S., Kwon H.J., Han Y.H. (2003). Peroxiredoxin II is essential for sustaining life span of erythrocytes in mice. Blood.

[44] Low F.M., Hampton M.B., Winterbourn C.C. (2008). Peroxiredoxin 2 and peroxide metabolism in the erythrocyte. Antioxid. Redox Signal..

[45] Kim K.M., Kim D.Y., Lee D.S., Kim J.W., Koh J.T., Kim E.J., Jang W.G. (2019). Peroxiredoxin II negatively regulates BMP2-induced osteoblast differentiation and bone formation via PP2A Calpha-mediated Smad1/5/9 dephosphorylation. Exp. Mol. Med..

[46] Yang H.Y., Jeong D.K., Kim S.H., Chung K.J., Cho E.J., Jin C.H., Yang U., Lee S.R., Lee D.S., Lee T.H. (2008). Gene expression profiling related to the enhanced erythropoiesis in mouse bone marrow cells. J. Cell. Biochem..

[47] Yang H.Y., Kwon J., Choi H.I., Park S.H., Yang U., Park H.R., Ren L., Chung K.J., Kim Y.U., Park B.J. (2012). In-depth analysis of cysteine oxidation by the RBC proteome: Advantage of peroxiredoxin II knockout mice. Proteomics.

[48] Kwon T.H., Han Y.H., Hong S.G., Lee D.J., Ha H.L., Kang S.W., Li W., Yoon D.Y., Yu D.Y. (2012). Reactive oxygen species mediated DNA damage is essential for abnormal erythropoiesis in peroxiredoxin II(-/-) mice. Biochem. Biophys. Res. Commun..

[49] Han Y.H., Kim S.U., Kwon T.H., Lee D.S., Ha H.L., Park D.S., Woo E.J., Lee S.H., Kim J.M., Chae H.B. (2012). Peroxiredoxin II is essential for preventing hemolytic anemia from oxidative stress through maintaining hemoglobin stability. Biochem. Biophys. Res. Commun..

[50] Nagababu E., Mohanty J.G., Friedman J.S., Rifkind J.M. (2013). Role of peroxiredoxin-2 in protecting RBCs from hydrogen peroxide-induced oxidative stress. Free Radic. Res..

[51] Matte A., Pantaleo A., Ferru E., Turrini F., Bertoldi M., Lupo F., Siciliano A., Ho Zoon C., De Franceschi L. (2014). The novel role of peroxiredoxin-2 in red cell membrane protein homeostasis and senescence. Free Radic. Biol. Med..

[52] Matte A., De Falco L., Iolascon A., Mohandas N., An X., Siciliano A., Leboeuf C., Janin A., Bruno M., Choi S.Y. (2015). The Interplay Between Peroxiredoxin-2 and Nuclear Factor-Erythroid 2 Is Important in Limiting Oxidative Mediated Dysfunction in beta-Thalassemic Erythropoiesis. Antioxid. Redox Signal..

[53] Matte A., De Falco L., Federti E., Cozzi A., Iolascon A., Levi S., Mohandas N., Zamo A., Bruno M., Lebouef C. (2018). Peroxiredoxin-2: A Novel Regulator of Iron Homeostasis in Ineffective Erythropoiesis. Antioxid. Redox Signal..

[54] Cho C.S., Yoon H.J., Kim J.Y., Woo H.A., Rhee S.G. (2014). Circadian rhythm of hyperoxidized peroxiredoxin II is determined by hemoglobin autoxidation and the 20S proteasome in red blood cells. Proc. Natl. Acad. Sci. USA.

[55] Johnson R.M., Ho Y.S., Yu D.Y., Kuypers F.A., Ravindranath Y., Goyette G.W. (2010). The effects of disruption of genes for peroxiredoxin-2, glutathione peroxidase-1, and catalase on erythrocyte oxidative metabolism. Free Radic. Biol. Med..

[56] Han Y.H., Kim H.S., Kim J.M., Kim S.K., Yu D.Y., Moon E.Y. (2005). Inhibitory role of peroxiredoxin II (Prx II) on cellular senescence. FEBS Lett..

[57] Li W., Febbraio M., Reddy S.P., Yu D.Y., Yamamoto M., Silverstein R.L. (2010). CD36 participates in a signaling pathway that regulates ROS formation in murine VSMCs. J. Clin. Investig..

[58] Kang D.H., Lee D.J., Lee K.W., Park Y.S., Lee J.Y., Lee S.H., Koh Y.J., Koh G.Y., Choi C., Yu D.Y. (2011). Peroxiredoxin II is an essential antioxidant enzyme that prevents the oxidative inactivation of VEGF receptor-2 in vascular endothelial cells. Mol. Cell.

[59] Kang D.H., Lee D.J., Kim J., Lee J.Y., Kim H.W., Kwon K., Taylor W.R., Jo H., Kang S.W. (2013). Vascular injury involves the overoxidation of peroxiredoxin type II and is recovered by the peroxiredoxin activity mimetic that induces reendothelialization. Circulation.

[60] Jang J.Y., Wang S.B., Min J.H., Chae Y.H., Baek J.Y., Yu D.Y., Chang T.S. (2015). Peroxiredoxin II is an antioxidant enzyme that negatively regulates collagen-stimulated platelet function. J. Biol. Chem..

[61] Yang Y., Bazhin A.V., Werner J., Karakhanova S. (2013). Reactive oxygen species in the immune system. Int. Rev. Immunol..

[62] Moon E.Y., Noh Y.W., Han Y.H., Kim S.U., Kim J.M., Yu D.Y., Lim J.S. (2006). T lymphocytes and dendritic cells are activated by the deletion of peroxiredoxin II (Prx II) gene. Immunol. Lett..

[63] Moon E.Y., Lee J.H., Oh S.Y., Ryu S.K., Kim H.M., Kwak H.S., Yoon W.K. (2006). Reactive oxygen species augment B-cell-activating factor expression. Free Radic. Biol. Med..

[64] Moon E.Y., Han Y.H., Lee D.S., Han Y.M., Yu D.Y. (2004). Reactive oxygen species induced by the deletion of peroxiredoxin II (PrxII) increases the number of thymocytes resulting in the enlargement of PrxII-null thymus. Eur. J. Immunol..

[65] Yang C.S., Lee D.S., Song C.H., An S.J., Li S., Kim J.M., Kim C.S., Yoo D.G., Jeon B.H., Yang H.Y. (2007). Roles of peroxiredoxin II in the regulation of proinflammatory responses to LPS and protection against endotoxin-induced lethal shock. J. Exp. Med..

[66] Park J.G., Yoo J.Y., Jeong S.J., Choi J.H., Lee M.R., Lee M.N., Hwa Lee J., Kim H.C., Jo H., Yu D.Y. (2011). Peroxiredoxin 2 deficiency exacerbates atherosclerosis in apolipoprotein E-deficient mice. Circ. Res..

[67] Federti E., Matte A., Ghigo A., Andolfo I., James C., Siciliano A., Leboeuf C., Janin A., Manna F., Choi S.Y. (2017). Peroxiredoxin-2 plays a pivotal role as multimodal cytoprotector in the early phase of pulmonary hypertension. Free Radic. Biol. Med..

[68] Won H.Y., Jang E.J., Lee K., Oh S., Kim H.K., Woo H.A., Kang S.W., Yu D.Y., Rhee S.G., Hwang E.S. (2013). Ablation of peroxiredoxin II attenuates experimental colitis by increasing FoxO1-induced Foxp3+ regulatory T cells. J. Immunol..

[69] Kang D.H., Lee D.J., Lee S., Lee S.Y., Jun Y., Kim Y., Kim Y., Lee J.S., Lee D.K., Lee S. (2017). Interaction of tankyrase and peroxiredoxin II is indispensable for the survival of colorectal cancer cells. Nat. Commun..

[70] Kang D.H., Lee J.H.S., Kang S.W. (2017). Survival of APC-mutant colorectal cancer cells requires interaction between tankyrase and a thiol peroxidase, peroxiredoxin II. BMB Rep..

[71] Park H., Noh A.L., Kang J.H., Sim J.S., Lee D.S., Yim M. (2015). Peroxiredoxin II negatively regulates lipopolysaccharide-induced osteoclast formation and bone loss via JNK and STAT3. Antioxid. Redox Signal..

[72] Kim S.U., Jin M.H., Kim Y.S., Lee S.H., Cho Y.S., Cho K.J., Lee K.S., Kim Y.I., Kim G.W., Kim J.M. (2011). Peroxiredoxin II preserves cognitive function against age-linked hippocampal oxidative damage. Neurobiol. Aging.

[73] Kim J.H., Park S.J., Chae U., Seong J., Lee H.S., Lee S.R., Lee S., Lee D.S. (2018). Peroxiredoxin 2 mediates insulin sensitivity of skeletal muscles through regulation of protein tyrosine phosphatase oxidation. Int. J. Biochem. Cell Biol..

[74] Park S.J., Kim J.H., Kim T.S., Lee S.R., Park J.W., Lee S., Kim J.M., Lee D.S. (2017). Peroxiredoxin 2 regulates PGF2alpha-induced corpus luteum regression in mice by inhibiting ROS-dependent JNK activation. Free Radic. Biol. Med..

[75] Park S.J., Kim J.H., Lee D.G., Kim J.M., Lee D.S. (2018). Peroxiredoxin 2 deficiency accelerates age-related ovarian failure through the reactive oxygen species-mediated JNK pathway in mice. Free Radic. Biol. Med..

[76] Li L., Shoji W., Takano H., Nishimura N., Aoki Y., Takahashi R., Goto S., Kaifu T., Takai T., Obinata M. (2007). Increased susceptibility of MER5 (peroxiredoxin III) knockout mice to LPS-induced oxidative stress. Biochem. Biophys. Res. Commun.

[77] Zambrowicz B.P., Friedrich G.A., Buxton E.C., Lilleberg S.L., Person C., Sands A.T. (1998). Disruption and sequence identification of 2,000 genes in mouse embryonic stem cells. Nature.

[78] Huh J.Y., Kim Y., Jeong J., Park J., Kim I., Huh K.H., Kim Y.S., Woo H.A., Rhee S.G., Lee K.J. (2012). Peroxiredoxin 3 is a key molecule regulating adipocyte oxidative stress, mitochondrial biogenesis, and adipokine expression. Antioxid. Redox Signal..

[79] Lee K.P., Shin Y.J., Cho S.C., Lee S.M., Bahn Y.J., Kim J.Y., Kwon E.S., Jeong D.Y., Park S.C., Rhee S.G. (2014). Peroxiredoxin 3 has a crucial role in the contractile function of skeletal muscle by regulating mitochondrial homeostasis. Free Radic. Biol. Med..

[80] Zhang Y.G., Wang L., Kaifu T., Li J., Li X., Li L. (2016). Featured Article: Accelerated decline of physical strength in peroxiredoxin-3 knockout mice. Exp. Biol. Med..

[81] Li L., Kaifu T., Obinata M., Takai T. (2009). Peroxiredoxin III-deficiency sensitizes macrophages to oxidative stress. J. Biochem..

[82] Bae S.H., Sung S.H., Lee H.E., Kang H.T., Lee S.K., Oh S.Y., Woo H.A., Kil I.S., Rhee S.G. (2012). Peroxiredoxin III and sulfiredoxin together protect mice from pyrazole-induced oxidative liver injury. Antioxid. Redox Signal..

[83] Li L., Shoji W., Oshima H., Obinata M., Fukumoto M., Kanno N. (2008). Crucial role of peroxiredoxin III in placental antioxidant defense of mice. FEBS Lett..

[84] Li L., Obinata M., Hori K. (2010). Role of peroxiredoxin III in the pathogenesis of pre-eclampsia as evidenced in mice. Oxid. Med. Cell. Longev..

[85] Baek J.Y., Park S., Park J., Jang J.Y., Wang S.B., Kim S.R., Woo H.A., Lim K.M., Chang T.S. (2017). Protective Role of Mitochondrial Peroxiredoxin III against UVB-Induced Apoptosis of Epidermal Keratinocytes. J. Investig. Dermatol..

[86] Yim S.H., Kim Y.J., Oh S.Y., Fujii J., Zhang Y., Gladyshev V.N., Rhee S.G. (2011). Identification and characterization of alternatively transcribed form of peroxiredoxin IV gene that is specifically expressed in spermatids of postpubertal mouse testis. J. Biol. Chem..

[87] Matsumoto A., Okado A., Fujii T., Fujii J., Egashira M., Niikawa N., Taniguchi N. (1999). Cloning of the peroxiredoxin gene family in rats and characterization of the fourth member. FEBS Lett..

[88] Okado-Matsumoto A., Matsumoto A., Fujii J., Taniguchi N. (2000). Peroxiredoxin IV is a secretable protein with heparin-binding properties under reduced conditions. J. Biochem..

[89] Iuchi Y., Okada F., Tsunoda S., Kibe N., Shirasawa N., Ikawa M., Okabe M., Ikeda Y., Fujii J. (2009). Peroxiredoxin 4 knockout results in elevated spermatogenic cell death via oxidative stress. Biochem. J..

[90] Takagi T., Homma T., Fujii J., Shirasawa N., Yoriki H., Hotta Y., Higashimura Y., Mizushima K., Hirai Y., Katada K. (2019). Elevated ER stress exacerbates dextran sulfate sodium-induced colitis in PRDX4-knockout mice. Free Radic. Biol. Med..

[91] Guo X., Noguchi H., Ishii N., Homma T., Hamada T., Hiraki T., Zhang J., Matsuo K., Yokoyama S., Ishibashi H. (2019). The Association of Peroxiredoxin 4 with the Initiation and Progression of Hepatocellular Carcinoma. Antioxid. Redox Signal..

[92] Zito E., Hansen H.G., Yeo G.S., Fujii J., Ron D. (2012). Endoplasmic reticulum thiol oxidase deficiency leads to ascorbic acid depletion and noncanonical scurvy in mice. Mol. Cell.

[93] Homma T., Kurahashi T., Lee J., Nabeshima A., Yamada S., Fujii J. (2018). Double Knockout of Peroxiredoxin 4 (Prdx4) and Superoxide Dismutase 1 (Sod1) in Mice Results in Severe Liver Failure. Oxid. Med. Cell. Longev..

[94] Kim M.H., Park S.J., Kim J.H., Seong J.B., Kim K.M., Woo H.A., Lee D.S. (2018). Peroxiredoxin 5 regulates adipogenesis-attenuating oxidative stress in obese mouse models induced by a high-fat diet. Free Radic. Biol. Med..

[95] Eppig J.T., Motenko H., Richardson J.E., Richards-Smith B., Smith C.L. (2015). The International Mouse Strain Resource (IMSR): Cataloging worldwide mouse and ES cell line resources. Mamm. Genome.

[96] Kim M.H., Seong J.B., Huh J.W., Bae Y.C., Lee H.S., Lee D.S. (2020). Peroxiredoxin 5 ameliorates obesity-induced non-alcoholic fatty liver disease through the regulation of oxidative stress and AMP-activated protein kinase signaling. Redox Biol..

[97] Wang X., Phelan S.A., Forsman-Semb K., Taylor E.F., Petros C., Brown A., Lerner C.P., Paigen B. (2003). Mice with targeted mutation of peroxiredoxin 6 develop normally but are susceptible to oxidative stress. J. Biol. Chem..

[98] Mo Y., Feinstein S.I., Manevich Y., Zhang Q., Lu L., Ho Y.S., Fisher A.B. (2003). 1-Cys peroxiredoxin knock-out mice express mRNA but not protein for a highly related intronless gene. FEBS Lett..

[99] Wang Y., Feinstein S.I., Manevich Y., Ho Y.S., Fisher A.B. (2006). Peroxiredoxin 6 gene-targeted mice show increased lung injury with paraquat-induced oxidative stress. Antioxid. Redox Signal..

[100] Wang Y., Feinstein S.I., Manevich Y., Ho Y.S., Fisher A.B. (2004). Lung injury and mortality with hyperoxia are increased in peroxiredoxin 6 gene-targeted mice. Free Radic. Biol. Med..

[101] Liu G., Feinstein S.I., Wang Y., Dodia C., Fisher D., Yu K., Ho Y.S., Fisher A.B. (2010). Comparison of glutathione peroxidase 1 and peroxiredoxin 6 in protection against oxidative stress in the mouse lung. Free Radic. Biol. Med..

[102] Fisher A.B., Dodia C., Feinstein S.I., Ho Y.S. (2005). Altered lung phospholipid metabolism in mice with targeted deletion of lysosomal-type phospholipase A2. J. Lipid Res..

[103] Fisher A.B., Dodia C., Sorokina E.M., Li H., Zhou S., Raabe T., Feinstein S.I. (2016). A novel lysophosphatidylcholine acyl transferase activity is expressed by peroxiredoxin 6. J. Lipid Res..

[104] Lien Y.C., Feinstein S.I., Dodia C., Fisher A.B. (2012). The roles of peroxidase and phospholipase A2 activities of peroxiredoxin 6 in protecting pulmonary microvascular endothelial cells against peroxidative stress. Antioxid. Redox Signal..

[105] Li H., Benipal B., Zhou S., Dodia C., Chatterjee S., Tao J.Q., Sorokina E.M., Raabe T., Feinstein S.I., Fisher A.B. (2015). Critical role of peroxiredoxin 6 in the repair of peroxidized cell membranes following oxidative stress. Free Radic. Biol. Med..

[106] Fisher A.B., Vasquez-Medina J.P., Dodia C., Sorokina E.M., Tao J.Q., Feinstein S.I. (2018). Peroxiredoxin 6 phospholipid hydroperoxidase activity in the repair of peroxidized cell membranes. Redox Biol..

[107] Wang Y., Feinstein S.I., Fisher A.B. (2008). Peroxiredoxin 6 as an antioxidant enzyme: Protection of lung alveolar epithelial type II cells from H2O2-induced oxidative stress. J. Cell. Biochem..

[108] Sundar I.K., Chung S., Hwang J.W., Arunachalam G., Cook S., Yao H., Mazur W., Kinnula V.L., Fisher A.B., Rahman I. (2010). Peroxiredoxin 6 differentially regulates acute and chronic cigarette smoke-mediated lung inflammatory response and injury. Exp. Lung Res..

[109] Wang X., An X., Wang X., Hu X., Bi J., Tong L., Yang D., Song Y., Bai C. (2019). Peroxiredoxin 6 knockout aggravates cecal ligation and puncture-induced acute lung injury. Int. Immunopharmacol..

[110] Yang D., Jin M., Bai C., Zhou J., Shen Y. (2017). Peroxiredoxin 6 suppresses Muc5ac overproduction in LPS-induced airway inflammation through H2O2-EGFR-MAPK signaling pathway. Respir. Physiol. Neurobiol..

[111] Vazquez-Medina J.P., Dodia C., Weng L., Mesaros C., Blair I.A., Feinstein S.I., Chatterjee S., Fisher A.B. (2016). The phospholipase A2 activity of peroxiredoxin 6 modulates NADPH oxidase 2 activation via lysophosphatidic acid receptor signaling in the pulmonary endothelium and alveolar macrophages. FASEB J..

[112] Chatterjee S., Feinstein S.I., Dodia C., Sorokina E., Lien Y.C., Nguyen S., Debolt K., Speicher D., Fisher A.B. (2011). Peroxiredoxin 6 phosphorylation and subsequent phospholipase A2 activity are required for agonist-mediated activation of NADPH oxidase in mouse pulmonary microvascular endothelium and alveolar macrophages. J. Biol. Chem..

[113] Sorrell S.L., Golder Z.J., Johnstone D.B., Frankl F.E.K. (2016). Renal peroxiredoxin 6 interacts with anion exchanger 1 and plays a novel role in pH homeostasis. Kidney Int..

[114] Kumin A., Schafer M., Epp N., Bugnon P., Born-Berclaz C., Oxenius A., Klippel A., Bloch W., Werner S. (2007). Peroxiredoxin 6 is required for blood vessel integrity in wounded skin. J. Cell. Biol..

[115] Asuni A.A., Guridi M., Sanchez S., Sadowski M.J. (2015). Antioxidant peroxiredoxin 6 protein rescues toxicity due to oxidative stress and cellular hypoxia in vitro, and attenuates prion-related pathology in vivo. Neurochem. Int..

[116] Eismann T., Huber N., Shin T., Kuboki S., Galloway E., Wyder M., Edwards M.J., Greis K.D., Shertzer H.G., Fisher A.B. (2009). Peroxiredoxin-6 protects against mitochondrial dysfunction and liver injury during ischemia-reperfusion in mice. Am. J. Physiol. Gastrointest. Liver Physiol..

[117] Roede J.R., Orlicky D.J., Fisher A.B., Petersen D.R. (2009). Overexpression of peroxiredoxin 6 does not prevent ethanol-mediated oxidative stress and may play a role in hepatic lipid accumulation. J. Pharmacol. Exp. Ther..

[118] Pacifici F., Arriga R., Sorice G.P., Capuani B., Scioli M.G., Pastore D., Donadel G., Bellia A., Caratelli S., Coppola A. (2014). Peroxiredoxin 6, a novel player in the pathogenesis of diabetes. Diabetes.

[119] Kuda O., Brezinova M., Silhavy J., Landa V., Zidek V., Dodia C., Kreuchwig F., Vrbacky M., Balas L., Durand T. (2018). Nrf2-Mediated Antioxidant Defense and Peroxiredoxin 6 Are Linked to Biosynthesis of Palmitic Acid Ester of 9-Hydroxystearic Acid. Diabetes.

[120] Melhem H., Spalinger M.R., Cosin-Roger J., Atrott K., Lang S., Wojtal K.A., Vavricka S.R., Rogler G., Frey-Wagner I. (2017). Prdx6 Deficiency Ameliorates DSS Colitis: Relevance of Compensatory Antioxidant Mechanisms. J. Crohns Colitis.

[121] Wang X., Phelan S.A., Petros C., Taylor E.F., Ledinski G., Jurgens G., Forsman-Semb K., Paigen B. (2004). Peroxiredoxin 6 deficiency and atherosclerosis susceptibility in mice: Significance of genetic background for assessing atherosclerosis. Atherosclerosis.

[122] Ozkosem B., Feinstein S.I., Fisher A.B., O’Flaherty C. (2015). Advancing age increases sperm chromatin damage and impairs fertility in peroxiredoxin 6 null mice. Redox Biol..

[123] Ozkosem B., Feinstein S.I., Fisher A.B., O’Flaherty C. (2016). Absence of Peroxiredoxin 6 Amplifies the Effect of Oxidant Stress on Mobility and SCSA/CMA3 Defined Chromatin Quality and Impairs Fertilizing Ability of Mouse Spermatozoa. Biol. Reprod..

[124] Fatma N., Singh P., Chhunchha B., Kubo E., Shinohara T., Bhargavan B., Singh D.P. (2011). Deficiency of Prdx6 in lens epithelial cells induces ER stress response-mediated impaired homeostasis and apoptosis. Am. J. Physiol..

[125] Fatma N., Kubo E., Sharma P., Beier D.R., Singh D.P. (2005). Impaired homeostasis and phenotypic abnormalities in Prdx6-/-mice lens epithelial cells by reactive oxygen species: Increased expression and activation of TGFbeta. Cell Death Differ..

[126] Kubo E., Hasanova N., Tanaka Y., Fatma N., Takamura Y., Singh D.P., Akagi Y. (2010). Protein expression profiling of lens epithelial cells from Prdx6-depleted mice and their vulnerability to UV radiation exposure. Am. J. Physiol..

[127] Rolfs F., Huber M., Gruber F., Bohm F., Pfister H.J., Bochkov V.N., Tschachler E., Dummer R., Hohl D., Schafer M. (2013). Dual role of the antioxidant enzyme peroxiredoxin 6 in skin carcinogenesis. Cancer Res..

[128] Burgio G. (2018). Redefining mouse transgenesis with CRISPR/Cas9 genome editing technology. Genome Biol..

